# Supplementary material for: Understanding the social drivers of antibiotic use during COVID-19 in Bangladesh: Implications for reduction of antimicrobial resistance
Source: PLoS One. 2021 Dec 14;16(12):e0261368. doi: 10.1371/journal.pone.0261368 (PMC8670684; doi:10.1371/journal.pone.0261368)
Supplement: S1 File — (DOCX) [file pone.0261368.s001.docx]

**Interview guide.**

**Section A: Demographic information**

Place of living:

Gender:

Age:

Marital status:

Education:

Occupation:

Monthly income (approximate):

Number of antibiotic courses taken in last 1 year:

Co-morbidities:

**Section B: History of illness**

- How are you feeling today?
- What were some health problems you were facing? When did those started? How long it took to get recovered?
- What did you do then (taking medicine by self or consultation with a physician)?

**Section C: Antibiotics taking history.**

- In your knowledge, in what purpose people should use antibiotics if they get COVID-19?
- In your opinion, what are some symptoms that require antibiotics for the COVID-19 patients?
- What type of treatment/measures did you take during your sickness to COVID-19 or COVID-19 like symptoms? (Details name, doses, days of course, why stop taking). Who suggested you to take those medicine? How did you consult with them?
- Could you please tell me in detail, why did you use antibiotic? What motivated you to take antibiotics?
- Who suggested you to use antibiotic for COVID-19/COVID-19 like symptoms? (Probe: did your doctor suggest to collect or what motivated you to collect?)
- (If doctor or someone else suggested) Why they suggested you to use antibiotics? Did they give any explanation?
- (If self-medication) Why did you take antibiotics for COVID? What were the things that motivated you to take antibiotics?
- Did your family members also get COVID-19 or have the suggestive symptoms of COVID-19? If yes, did they take antibiotics? If yes, why they took antibiotics?
- How did you collect/get antibiotic medicine for COVID-19? (Probe: self or someone else? Did the dispensary people ask you (or who bought) to show the prescription?
- How did you use antibiotics? (Probe: days of course, dose completion or not, if not why stop taking).

**Thank you for your time.**
