# Supplementary material for: Understanding the social drivers of antibiotic use during COVID-19 in Bangladesh: Implications for reduction of antimicrobial resistance
Source: PLoS One. 2021 Dec 14;16(12):e0261368. doi: 10.1371/journal.pone.0261368 (PMC8670684; doi:10.1371/journal.pone.0261368)
Supplement: S2 File — (DOCX) [file pone.0261368.s002.docx]

Supplementary file: COREQ (Consolidated criteria for Reporting Qualitative research) Checklist.

| **Item no** | **Domain/Topic** | **Description** | **Response/ Reported on page no.** |
| --- | --- | --- | --- |
| **Domain 1: Research team and reﬂexivity** | | | |
| ***Subdomain: Personal characteristics*** | | | |
| 1 | Interviewer/facilitator | Which author/s conducted the interview or focus group? | 7 |
| 2 | Credentials | What were the researcher’s credentials? E.g., PhD, MD | MSS, PhD, MD. |
| 3 | Occupation | What was their occupation at the time of the study? | Research, Teaching, and Clinical practice. |
| 4 | Gender | Was the researcher male or female? | Female-2, Male 5 |
| 5 | Experience and training | What experience or training did the researcher have? | Anthropology, Sociology, One Health, Qualitative, Infectious Disease |
| ***Subdomain: Relationship with Participants*** | | | |
| 6 | Relationship established | Was a relationship established prior to study commencement? | 7 |
| 7 | Participant knowledge of  the interviewer | What did the participants know about the researcher? e.g., personal goals, reasons for doing the research | 7 |
| 8 | Interviewer characteristics | What characteristics were reported about the inter viewer/facilitator? e.g., Bias, assumptions, reasons and interests in the research topic | 7 |
| **Domain 2: Study design** | | | |
| ***Subdomain: Theoretical framework*** | | | |
| 9 | Methodological orientation and Theory | What methodological orientation was stated to underpin the study? e.g., grounded theory, discourse analysis, ethnography, phenomenology, content analysis | 6 |
| ***Subdomain: Participant selection*** | | | |
| 10 | Sampling | How were participants selected? e.g., purposive, convenience, consecutive, snowball | 7 |
| 11 | Method of approach | How were participants approached? e.g., face-to-face, telephone, mail, email | 7 |
| 12 | Sample size | How many participants were in the study? | 7 |
| 13 | Non-participation | How many people refused to participate or dropped out? Reasons? | 7 |
| Subdomain: Setting | | | |
| 14 | Setting of data collection | Where was the data collected? e.g., home, clinic, workplace | 7 |
| 15 | Presence of non-participants | Was anyone else present besides the participants and researchers? | 9 |
| 16 | Description of sample | What are the important characteristics of the sample? e.g., demographic  data, date | 10-11 |
| ***Subdomain: Data collection*** | | | |
| 17 | Interview guide | Were questions, prompts, guides provided by the authors? Was it pilot tested? | 8 |
| 18 | Repeat interviews | Were repeat inter views carried out? If yes, how many? | No |
| 19 | Audio/visual recording | Did the research use audio or visual recording to collect the data? | 8 |
| 20 | Field notes | Were ﬁeld notes made during and/or after the interview or focus group? | No |
| 21 | Duration | What was the duration of the inter views or focus group? | 8 |
| 22 | Data saturation | Was data saturation discussed? | 7 |
| 23 | Transcripts returned | Were transcripts returned to participants for comment and/or correction? | It was not feasible. |
| **Domain 3: analysis and ﬁndings** | | | |
| ***Subdomain: Data analysis*** | | | |
| 24 | Number of data coders | How many data coders coded the data? | 7 |
| 25 | Description of the coding  tree | Did authors provide a description of the coding tree? | 11-13 |
| 26 | Derivation of themes | Were themes identiﬁed in advance or derived from the data? | 8 |
| 27 | Software | What software, if applicable, was used to manage the data? | 8 |
| 28 | Participant checking | Did participants provide feedback on the ﬁndings? | It was not feasible. |
| ***Subdomain: Reporting*** | | | |
| 29 | Quotations presented | Were participant quotations presented to illustrate the themes/ﬁndings? Was each quotation identiﬁed? e.g., participant number | 13-23 |
| 30 | Data and ﬁndings consistent | Was there consistency between the data presented and the ﬁndings? | 13-23 |
| 31 | Clarity of major themes | Were major themes clearly presented in the ﬁndings? | 13-23 |
| 32 | Clarity of minor themes | Is there a description of diverse cases or discussion of minor themes? | 18-19 |

Developed from: Tong A, Sainsbury P, Craig J. Consolidated criteria for reporting qualitative research (COREQ): a 32-item checklist for interviews and focus groups. International Journal for Quality in Health Care. 2007. Volume 19, Number 6: pp. 349 – 357
